# Clinical spectrum of early onset “Mediterranean” (homozygous p.P131L mutation) mitochondrial neurogastrointestinal encephalomyopathy

**DOI:** 10.1002/jmd2.12315

**Published:** 2022-07-10

**Authors:** Sema Kalkan Uçar, Havva Yazıcı, Ebru Canda, Esra Er, Fatma Derya Bulut, Cenk Eraslan, Hüseyin Onay, Bridget Elizabeth Bax, Mahmut Çoker

**Affiliations:** ^1^ Department of Pediatrics, Division of Metabolism and Nutrition Ege University Medical Faculty Izmir Turkey; ^2^ Department of Pediatrics, Division of Metabolism and Nutrition Çukurova University Medical Faculty Adana Turkey; ^3^ Department of Radiology, Division of Neuroradiology Ege University Medical Faculty Bornova Turkey; ^4^ Department of Genetics Ege University Medical Faculty Izmir Turkey; ^5^ Institute of Molecular and Clinical Sciences St George's University of London London UK

**Keywords:** clinical overview, mitochondrial neurogastrointestinal encephalomyopathy, thymidine phosphorylase

## Abstract

Mitochondrial neurogastrointestinal encephalomyopathy (MNGIE) is an autosomal recessive mitochondrial disorder characterized by cumulative and progressive gastrointestinal and neurological findings. This retrospective observational study, aimed to explore the time of presentation, diagnosis and clinical follow‐up of 13 patients with a confirmed MNGIE disease of Mediterranean origin. The mean age of symptom onset was 7 years (6 months−21 years) and the average diagnosis age was 15.4 years ±8.4. Four of 13 patients (30%) died before 30 years at the mean age of 19.7 years ±6.8. Cachexia and gastrointestinal symptoms were observed in all patients (100%). The mean body mass index standard deviation score at diagnosis was 4.8 ± 2.8. At least three subocclusive episodes were presented in patients who died in last year of their life. The main neurological symptom found in most patients was peripheral neuropathy (92%). Ten patients (77%) had leukoencephalopathy and the remaining three patients without were under 10 years of age. The new homozygous “Mediterranean” *TYMP* mutation, p.P131L (c.392 C > T) was associated with an early presentation and poor prognosis in nine patients (69%) from five separates families. Based on the observations from this Mediterranean MNGIE cohort, we propose that the unexplained abdominal pain combined with cachexia is an indicator of MNGIE. High‐platelet counts and nerve conduction studies may be supportive laboratory findings and the frequent subocclusive episodes could be a negative prognostic factor for mortality. Finally, the homozygous p.P131L (c.392 C > T) mutation could be associated with rapid progressive disease with poor prognosis.

**SYNOPSIS**

This is the first study to report about the clinical course and prognosis mitochondrial neurogastrointestinal encephalomyopathy patients with early onset Mediterranean homozygous p.P131L mutation. We share here our experience‐based recommendations on disease diagnosis, clinical findings, prognosis, and the main predictors of disease progression.

## 1 INTRODUCTION

Mitochondrial neurogastrointestinal encephalomyopathy (MNGIE, MIM: 603041) is a ultra‐rare, progressive autosomal recessive metabolic disorder caused by mutations in the nuclear gene *TYMP* (OMIM: 131222), which codes the enzyme thymidine phosphorylase (TP, EC: 2.4.2.4).^1^ Impairment of this enzyme results as reduced enzyme activity leading to accumulation of thymidine (dThd) and deoxyuridine (dUrd) levels in tissues and body fluids, and subsequently deoxyribonucleotide pool imbalance and mitochondrial‐DNA instability, including mitochondrial genome deletion and depletion and point mutations.^2^

The onset of disease is generally in the first and second decade of life, although the earliest reported age of onset is 5 months of age,^3^ and as late as the fifth decade.^4^ The natural history of the disease is still uncharacterized, however gastrointestinal symptoms, peripheral neuropathy, leukoencephalopathy, progressive external ophthalmoplegia, ptosis, sensorineural hearing loss, myopathy, and endocrine dysfunctions are reported as the main clinical domains. The disease is progressive and leads to death at an average age of 37.5 years.^3^, ^5^

The estimated prevalence of MNGIE is evaluated to be 0.1 /100000 (European data),^6^ with approximately 200 patients reported world‐wide.^1^ Low‐disease prevalence and multiple organ system involvement creates a complex clinical picture, causing diagnosis delays reported to be between 5 and 10 years.^7^

Unfortunately, there are currently only several experimental therapeutic approaches for the management of the disease. Temporary correction of the plasma dThd and dUrd concentrations can be achieved by conventional hemodialysis^8^, ^9^ and erythrocyte encapsulated thymidine phosphorylase infusions.^10^ Hematopoetic stem cell transplantation (HCST)^11^ and orthotopic liver transplantation^12^, ^13^ can halt disease progression through permanent restoration of the TP enzyme function, but morbidity for both^1^ and mortality for HCST^14^ were reported.

Here, we report the time of presentation and diagnosis, emphasize the family screening, and expand the natural history and outcomes from the clinical follow‐up patients with the MNGIE of Mediterranean origin.

## 2 METHOD

### 2.1 Subjects

This is a retrospective observational study designed in accordance with the current revision of the Helsinki Declaration and includes patients with MNGIE who were followed at the Ege University Faculty of Medicine, Department of Pediatrics, Division of Pediatric Metabolism and Nutrition for the past 10 years (June 2012–March 2022). The study was approved by local ethical committee (E‐99166796‐0500604‐577 754/22–2.1 T/37) and informed concert was obtained from each participant.

The inclusion criteria were a clinical diagnosis of MNGIE confirmed by *TYMP* gene sequencing of patients living in the Mediterranean area of Turkey. No further testing was conducted on patients with homozygous or compound heterozygous allelic pathogenic variants, however if one variant of uncertain significance or a wild‐type sequence was identified, the dThd and dUrd levels were measured. Sequence variants were analyzed by VarSome and classified according to the American College of Medical Genetics and Genomics guidelines.

The demographic, clinical, biochemical and neuroimaging findings of MNGIE patients are reported. Evaluation of peripheral neuropathy was performed by quantitative vibration, thermal discrimination thresholds, and nerve conduction studies. Age and gender‐specific normal ranges were applied when interpreting results.

### 2.2 Statistical analysis

Data analysis was performed with the statistical package SPSS Inc., version 21.0, (Chicago, IL, USA). Continuous variables are displayed as arithmetical means plus or minus SD; categorical variables are displayed as frequencies or percentages. The Mann–Whitney/ Wilcoxon 2‐sample test (Kruskal‐Wallis test for two groups) was used when the analysis of variance test was not appropriate. A *p* level ≤0.05 determined statistical significance.

## 3 RESULTS

### 3.1 Demographic data

**FIGURE 1:**
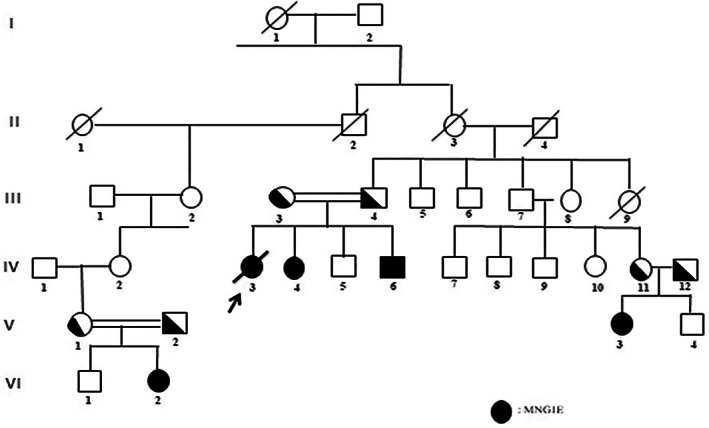
Patient pedigree, arrow indicates proband

(Table 1) thirteen patients (seven males, six females) were included in this study. The current median/mean age of our patients was 16.1 years (range 2–34)/17.7 ± 8.9 years. All patients, except one, had consanguineous parentage. Five patients were from the same family (Figure 1). The median/mean age of the diagnosis was 15.2 years (range 1.2–31.3)/15.4 ± 8.4 years. Four of our 13 patients (30%) died before 30 years at a mean age of 19.7 ± 6.8 years. The mean age of onset of the symptoms was 7 years (range 6 months −21 years).

**TABLE 1:** Demographic data of MNGIE patients

	Age at onset (years)	Age at diagnosis (years)	Gender	Current age (years)	Consanguinity	BMI (at diagnosis)	Weight (at diagnosis) (kg/SDS)	Weight change (follow‐up duration)	Height (at diagnosis) (cm/SDS)
P1	ND	12	M	Died at 16.3	+	10.54 kg/m^2^ (−4.77 SDS)	19.5 kg (−4.04 SDS)	ND	136 cm (−2.04 SDS)
P2	5	18	M	24.6	+	10.94 kg/m^2^ (−10.3 SDS)	28 kg (−8.35 SDS)	30 kg (30 months) (−7.62 SDS)	160 cm (−2.63 SDS)
P3	19	31.3	M	34.1	−	13.50 kg/m^2^ (−6.28 SDS)	42.3 kg (−4.27 SDS)	40.2 kg (9 months) (−3.55 SDS)	177 cm (0.13 SDS)
P4	6	28	F	Died at 30.5	+	15.70 kg/m^2^ (−4.58 SDS)	39.2 kg (−3.75 SDS)	32.3 kg (15 months) (−5.73 SDS)	158 cm (−0.87 SDS)
P5^a^	15	20	F	Died at 20	+	14,17 kg/m^2^ (−6.6 SDS)	40 kg (−3.55 SDS)	ND	168 cm (0.83 SDS)
P6^a^	14	16.6	F	19.9	+	14.29 kg/m^2^ (−5.16 SDS)	37 kg (−3.61 SDS)	32.2 kg (52 months) (−5.76 SDS)	162 cm (−0.1 SDS)
P7^a^	9	9.8	M	12.8	+	15,09 kg/m^2^ (−0.97 SDS)	30 kg (−0,24 SDS)	40.7 kg (37 months) (−0.85 SDS)	141 cm (0.84 SDS)
P8^a^	2	3.7	F	5.1	+	13.29 kg/m^2^ (−2.01 SDS)	12.5 kg (−1.65 SDS)	16.9 kg (28 months) (−1.33 SDS)	97 cm (−0.74 SDS)
P9^a^	1	1.2	F	2.4	+	15.06 kg/m^2^ (−1.13 SDS)	8 kg (−1.11 SDS)	12.5 kg (27 months) (−1.40 SDS)	76 cm (−0.6 SDS)
P10	1	12.8	M	14.8	+	9.59 kg/m^2^ (−5.83 SDS)	18 kg (−4.64 SDS)	28.2 kg (28 months) (−4.95 SDS)	137 cm (−2.47 SDS)
P11	2	10.8	M	Died at 12	+	12,79 kg/m^2^ (−2.82 SDS)	24 kg (−2.37 SDS)	29.8 kg (22 months) (−3.00 SDS)	137 cm (−0.88 SDS)
P12	0.5	18	M	19.1	+	16.44 kg/m^2^ (−3.43 SDS)	51.5 kg (−2.57 SDS)	40 kg (19 months) (−4.79 SDS)	177 cm (0.13 SDS)
P13	7	18.4	M	18.7	+	12.6 kg/m^2^ (−9.35 SDS)	31.5 kg (−5.99 SDS)	25.5 kg (8 months) (−8.28 SDS)	158 cm (−0.87 SDS)
Mean ±SDS	7.0 ±6.5	15.4 ±8.4		17.7 ±8.9		13.3 kg/m^2^ ±2.0	29.3 kg ±12.6	28.3 months ±9.2	144.9 cm ±29.8

^a^ From the same family.

Abbreviations: F, Female; M, Male; ND, not determined.

### 3.2 Clinical data

(Table 2) cachexia was observed in all patients (100%). The mean body mass index standard deviation score (BMI‐SDS) at diagnosis was 4.8 ± 2.8 at a mean age of 15.2 years. For patients diagnosed before 10 years of age, e BMI‐SDS was −1.4 ± 0.5, with this decreasing to −5.9 ± 2.6 and − 5.8 ± 2.2 respectively, for patients diagnosed between 10 and 20 years and after 20 years.

**TABLE 2:** Clinical data of MNGIE patients

Patient number	Gastrointestinal findings	Hepatic findings	Neurological findings	Oculer findings	Other
Patient 1	Diarrhea, abdominal pain, vomiting, protein lossing enteropathy, feeding intolerance, requiring PEG	None	Demyelinating polyneuropathy, muscle weakness, MRI: cerebellar atrophy, T2‐hyperintensity of the white matter	Ptosis, ophthalmoplegia	Subaortic fibromuscular ridge
Patient 2	Abdominal pain, vomiting, diarrhea, cachexia	None	Demyelinating polyneuropathy, muscle weakness, MRI: cerebellar atrophy, T2‐hyperintensity of the white matter	Ptosis ophthalmoplegia	Hearing loss
Patient 3	Diarrhea, borborygmi, abdominal pain, protein lossing enteropathy	Hepatomegaly, hepatosteatosis	Demyelinating polyneuropathy, T2‐hyperintensity of the white matter	None	Osteopenia, arthritis, mild mitral regurgitation and tricuspid regurgitation
Patient 4	Vomiting, diarrhea, abdominal distention, abdominal pain, intestinal pseudo‐obstruction	Hepatomegaly, hepatosteatosis	Demyelinating polyneuropathy, MRI: cerebellar atrophy, T2‐hyperintensity of the white matter	Ptosis, ophthalmoplegia	Hyperglycemia, mild tricuspid regurgitation
Patient 5	Vomiting, diarrhea, abdominal distention, abdominal pain, intestinal pseudo‐obstruction	Hepatomegaly, hepatosteatosis	Demyelinating polyneuropathy, muscle weakness, MRI: cerebellar atrophy, T2‐hyperintensity of the white matter	Ptosis, ophthalmoplegia, Chorioretinal atrophy	Hearing loss, mild mitral regurgitation, and tricuspid regurgitation, hyperglycemia (insülin dependent)
Patient 6	Vomiting, diarrhea, abdominal distention, abdominal pain	Hepatomegaly, hepatosteatosis	Demyelinating polyneuropathy, muscle weakness, MRI: cerebellar atrophy, T2‐hyperintensity of the white matter	Ptosis, ophthalmoplegia	mitral valve prolapse, hyperglycemia (insülin dependent)
Patient 7	Borborygmi, abdominal pain	None	Demyelinating sensorimotor polyneuropathy	Ptosis, ophthalmoplegia	
Patient 8	Vomiting, abdominal pain	None	Demyelinating sensorimotor polyneuropathy	Peripapillary atrophy	None
Patient 9	Vomiting, diarrhea	None	None	None	Hearing loss
Patient 10	Vomiting, diarrhea, intestinal pseudo‐obstruction, feeding intolerance, TPN dependent, abdominal pain	Hepatomegaly, hepatosteatosis	Muscle weakness, sensorimotor peripheral neuropathy, T2‐hyperintensity of the white matter	Retinitis pigmentosa, chorioretinal atrophy	Hearing loss, osteopenia, mitral valve prolapse, mild mitral regurgitation, and tricuspid regurgitation
Patient 11	Abdominal pain, distention, diarrhea, vomiting, nausea	None	Hyporeflexia, sensorimotor peripheral neuropathy, T2‐hyperintensity of the white matter	Retinal pigmentary changes	
Patient 12	Abdominal pain, distention, diarrhea, vomiting, nausea	Chirrhosis, hepatosteatosis, hepatomegaly	Hyporeflexia, sensorimotor peripheral neuropathy, T2‐hyperintensity of the white matter	Ptosis	Hyperglycemia, osteopenia
Patient 13	Vomiting, diarrhea, intestinal pseudo‐obstruction	Hepatosteatosis	Hyporeflexia, sensorimotor peripheral neuropathy, T2‐hyperintensity of the white matter	None	None

Gastrointestinal symptoms were reported in all patients (100%). The specific abdominal pain, diarrhea, vomiting, and intestinal pseudoobstruction were observed in 11 (85%), 12 (92%), 11 (85%) and 4 (30%) patients, respectively. The subocclusive episodes were reported in four patients who died before following up. Patients P1, P4, P5, and P10 experienced at least three episodes of pseudoobstruction in the last year of their life. Hepatomegaly was found in 5 (38%) patients and 7 (54%) patients had hepatosteatosis.

Neurological symptoms such as peripheral neuropathy, was observed in almost all our patients 12 (92%). The only patient who did not have a peripheral neuropathy was the youngest patient (P 9, current age: 2 years and 4 months). Peripheral neuropathy manifestations included limb weakness in 5 (38%) and as ptosis in 7 (54%) patients. Peripheral neuropathy was confirmed by nerve conduction studies performed in all patients. Hearing loss was found in 4 (30%) patients. Ten patients (77%) had leukoencephalopathy, detected by cranial magnetic resonance imaging (MRI). The remaining three patients who did not have leukoencephalopathy (P7, P8, and P9) were under 10 years of age.

Cardiac manifestations were observed in 6 (46%) patients. Mitral valves prolapse was shown by echocardiography (ECHO) in two patients who had systolic heart murmurs. Five patients had both mild mitral regurgitation and tricuspid regurgitation and one patient had subaortic fibromuscular ridge.

### 3.3 Biochemical and laboratory data

The mean platelet count of the patients was at the upper range of 351 461 ± 123 541/μl, with five patients (38%) having counts greater than 450 000/μl (thrombocytosis) (Table 3).

**TABLE 3:** Biochemical and genetic analyis of 13 patients diagnosed with MNGIE

Patient No	Hemoglobin (g/dl) (*N*: 11.7–15.5)	Platelet (10^3^/μl) (*N*: 150–450)	Calcium (mEq/dl) (*N*: 8.6–10.2)	Glucose (mg/dl) (*N*: 60–110)	Insulin (mU/L) (*N*: 2.6–10)	AST (U/L) (*N*: <31)	ALT (U/L) (*N*: <34)	Lactate (mg/dl) (*N*: 5–20)	Ammonia (μg/dl) (*N*: 17–80)	Creatine (mg/dl) (*N*: 0.3–1.1)	Uric acid (mg/dl) (2.0–5.5)	LDL (mg/dl) (*N*: <130)	Trigly ceride (mg/dl) (*N*: <130)	Thymidine (μmol/L) (≤0.05 μmol/L)	2'‐Deoxy uridine (μmol/L) (≤0.05 μmol/L)	*TYMP* mutation analysis
P1	12.4	172 000	8	ND	ND	40	23	ND	ND	0.2	1.4	ND	ND	ND	ND	Homozygous p.P131L (c.392 C > T)
P2	14.8	257 000	9.1	92	ND	29	20	22.5	57	0.58	4.7	74	107	11.4	17.0	Homozygous c. 978_979 İnsC
P3	10.8	608 000	8	86	11.3	20	13	18.3	29	0.34	3.5	36	141	16.64	21.11	Homozygous p.L347P (c.1040 T > C)
P4	10.8	295 000	9.2	103	2.91	15	15	14.9	37	0.56	4.4	14	228	9.83	12.80	Homozygous p.G428R (c.1282G > C)
P5^a^	14	477 000	9.7	136	171	35	34	43	ND	0.33	3.9	62	307	ND	ND	Homozygous p.P131L (c.392 C > T)ᶲ
P6^a^	15.6	315 000	8.8	206	76.4	73	55	15.5	53	0.32	4.9	56	1007	0.17	0.44	Homozygous p.P131L (c.392 C > T)ᶲ
P7^a^	13.7	317 000	10.2	75	6.8	30	14	16.2	26	0.42	3.6	113	62	11.94	11.21	Homozygous p.P131L (c.392 C > T)^a^
P8^a^	13.9	203 000	9.3	126	28	37	13	28.2	56	0.31	2.3	73	103	ND	ND	Homozygous p.P131L (c.392 C > T)^a^
P9^a^	11.7	464 000	10.5	87	3.2	35	12	41	ND	0.2	3.9	43	262	ND	ND	Homozygous p.P131L (c.392 C > T)^a^
P10	14.4	360 000	9.4	80	2.1	27	18	23	43	0.29	1.3	39	152	8.54	9.86	Homozygous p.P131L (c.392 C > T)
P11	14.5	403 000	9.8	58	2.65	29	16	16	34	0.42	4.9	35	68	ND	ND	Homozygous p.P131L 1(c.392 C > T)
P12	16.1	256 000	9.8	124	102	55	71	24.4	47	0.57	4.9	37	128	ND	ND	Homozygous p.P131L (c.392 C > T)
P13	9.7	442 000	9.1	82	4.7	44	32	39	43	0.18	1.9	60	199	ND	ND	Homozygous p.G72E (c.215G > A)
Mean ± SDS	13.2 ± 1.9	351 461 ± 123 541	9.3 ± 0.74	104.6 ± 39.4	37.4 ± 55.8	36.1 ± 15.1	25.8 ± 18.2	25.2 ± 10.4	42.5 ± 10.9	0.36 ± 0.14	3.5 ± 1.3	53.5 ± 25.7	230.3 ± 256.2	9.7 ± 5.4	12.1 ± 7.1	

Abbreviations: AST, aspartate aminotransferase; ALT, alanine aminotransferase; LDL, lactate dehydrogenase; ND, not determined.

^a^ From the same family.

Slightly elevated lactate levels were determined in 7 (54%) patients with a mean level of 25.2 ± 10.4 mg/dl. Increased triglyceride levels and all other laboratory features are reported in Table 3. The plasma concentrations of dThd and dUrd levels were also reported for six patients. Only P2 had TP activity measurement (TP: 0 nmol thymine/h/[mg protein]).

Nine patients (69%) (five from the same family) carried the new p.P131L (c.392 C > T) *TYMP* mutation. The other four patients were homozygous for p.L347P (c.1040 T > C), p.G428R (c.1282G > C), p.G72E (c.215G > A) and c.978_979ins C. The molecular analyses of *TYMP* are provided in Table 3. All patients, except one, had consanguineous parentage.

Three patients who were homozygous for the p.P131L *TYMP* mutation died at the ages of 12, 16 and 20 years, and another patient homozygous for p.G428R died at 30.5 years of age.

### 3.4 Follow‐up

Allogeneic HSCT (AHSCT) was performed in four patients. Patients 6, 10, 12, and 13 were followed up at 25, 12, 6, and 3 months after AHSCT, respectively. Patient 10 and 12 died from severe GI complications. AHSCT follow‐up and the nutritional support of MNGIE patients monitored for the metabolic effects, plus the possible mechanisms of action on mitochondrial energy homeostasis will be discussed in separate papers.

### 3.5 Pregnancy

Prior to diagnosis, one patient (P4) was delivered by cesarean of a healthy boy with a birth weight of 3300 grams. The pregnancy was reported to be uneventful. The patient reported to be clinically improved during pregnancy, with a weight gain of around 8 kg and reduced GI symptoms.

## 4 DISCUSSION

In our cohort of 13 Mediterranean MNGIE patients with a predominance of the homozygous p.P131L mutation, an early onset of the initial symptoms, particularly cachexia and unexplained abdominal pain were reported. Additionally, slightly higher platelet and lactate levels, peripheral neuropathy were detected in almost all patients and a higher and early mortality was observed.

The mean age of onset of the symptoms in our patients was 7 years (6 months–21 years). This contrasts with the mean age at onset reported as 17.9 years (5 months–43 years) in the position statements on the prognosis of MNGIE.^1^ Previous papers report disease onset between the first and second and decade of life, with an average age of 18.5 years.^3^, ^15^ The earliest reported age of onset was 5 months of age.^3^ One of our patients had disease onset at 6 months of age. Although, the first manifestation symptoms and severity can vary among patients, and even within the same family, we conclude that our patients have an early presentation compared to the classical early onset phenotype.

A further important observation was that the mean age of the diagnosis in our cohort was 15.4 years ±8.4, which means at least 8 years of delay before diagnosis. The literature well documents that most patients with MNGIE are misdiagnosed with malabsorption, Crohn's disease, inflammatory bowel disease or anorexia nervosa and diagnosis delays of up to 10 years.^8^, ^16^, ^17^, ^18^

According to the latest position paper of MNGIE by Hirano et al.,^1^ the mean age at death was reported to range between 35 and 37 years, with a and survival before 19 years being around 100% and then decreasing to <5% after 50 years. The disease is progressive and causes death at an average age of 37.6 years, with a range of 26–58 years.^2^ Common causes of death are cachexia, metabolic acidosis, pneumonia, peritonitis, and complications of bacterial overgrowth.^2^, ^19^, ^20^ However, in our Mediterranean MNGIE patients cohort, the age of death was much earlier at 19.7 years ±6.8. Three of the four deceased patients had the homozygous mutation p.P131L. The duration between their diagnosis and death ranged between 1 and 4 years. These data strongly support the aggressive progression of the Mediterranean MNGIE patients.

The cachexia, particularly with normal and /or increased nutritional intake, is an important finding of MNGIE patients.^2^, ^19^, ^20^ Weight loss was presented in all our patients at the diagnosis time. We noticed that the younger the patients are; the less weight loss was observed. Following diagnosis, aggressive nutritional support was able to diminish the weight loss in most of our patients, but still presented after 28 ± 9.2 months of follow‐up. Despite the absence of literature supporting the prognostic role of weight loss, malnutrition should be considered as a negative prognostic factor and is an important cause of mortality for patents with MNGIE. We observed a dramatic weight loss prior to the death of the four deceased patients described in our cohort, with patient 4 providing the best example.

The gastrointestinal dysfunction in MNGIE is well known and described. Abdominal pain, abdominal distention, diarrhea, dysphagia and pseudo obstruction are the main findings.^1^, ^7^, ^19^ The diarrhea, abdominal pain and vomiting were the most frequent gastrointestinal manifestations observed in our patients (92%, 92%, and 83%, respectively) in parallel with the literature. The otherwise unexplained abdominal pain combined with cachexia should be an important clinical clue to indicate MNGIE. In our cohort the subocclusive episodes presented in patients who died. From a clinical standpoint, intestinal subocclusive episodes are characterized by intestinal pseudo‐obstruction attacks.^21^ They are caused by marked dysfunction of gut propulsive motility, mimicking mechanical obstruction in a clinical picture. Patients are usually presented with severe abdominal pain and distension, bloating and vomiting (at least 20–25 times/day). Generally, these episodes are accompanied with constipation. However, particularly before and after; sometimes during the attacks a severe diarrhea is a very important part of the clinical status. This is a highly morbid and often life‐threatening condition of chronic intestinal failure.^21^ Therefore, we would like to highlight the presence of subocclusive episodes to be a sign of the severity of GI involvement and to be considered as a negative prognostic factor for mortality.

Patients presenting hepatopathies such as hepatic steatosis, hepatomegaly, increased transaminases, and cirrhosis, have also been reported.^3^, ^22^, ^23^ Consistent with these findings organomegaly was noted in 5 (38%) patients.

Neurological features suggesting peripheral neuropathy are one of the cardinal clues about MNGIE. Twelve (92%) of our patients had peripheral neuropathy detected by nerve conduction studies. The high presence of peripheral neuropathy in our cohort is supported by the literature.^1^, ^3^, ^18^, ^19^ Therefore, in patients with cachexia and abdominal pain, the non‐invasive procedure such as nerve conduction studies should be considered in cases suggestive of MNGIE disease. On the other hand, the neurological symptoms such as ptosis and hearing could also support the possible diagnosis of MNGIE. As with our findings (30%), the study of Garone et al.^3^ reported that 39% of patients presented with hearing loss. Ocular symptoms including ptosis are also common neurological findings in patients with this condition.^24^ Similarly, ptosis were observed in 7 (58%) patients. Another important neurological finding is progressive leukoencephalopathy which is almost universally present in patients with MNGIE.^3^ Ten (77%) of our patients had leukoencephalopathy, detected by cranial MRI. Interestingly, the three patients who did not have leukoencephalopathy (P7–P8–P9) were under 10 years of age. We speculated that the progressive nature of neurological findings in MNGIE may start with peripheral (gastrointestinal/neuroaxonal) and finalize with central nervous system involvement.

Cardiac manifestations are usually asymptomatic in MNGIE although there are reports of occasional cardiac complications, including a prolonged QT interval, cardiac arrest and supraventricular tachycardia.^3^, ^25^ Abnormal ECG has also been reported in some individuals.^26^ Cardiac dysfunction in affected twins with mitral valve prolapse and systolic heart murmurs was described in one study.^22^ Two of our patients had mitral valve prolapse, as demonstrated by ECHO.

Increased triglyceride levels and elevated plasma lactate in our cohort are consistent with the literature.^26^, ^27^ It is well known that some MNGIE patients display endocrine and metabolic dysfunctions, such as endocrine/exocrine pancreatic insufficiency, diabetes, and glucose intolerance.^3^ Elevated plasma lipids were also observed in our cohort. Impairment of mitochondrial function may lead to the lipid accumulation and insulin resistance.^28^ One of our patients had severe hypertriglyceridemia (1007 mg/dl), elevated insulin level (76.4 μIU/ml) and insulin resistance (HOMA‐IR: 38.8).

An interesting finding was the slightly elevated level platelet count detected in our patients and the fact that 38% of patients had counts greater than 450 000/μl. P3 had the highest platelet level as well as the highest dThd and dUrd levels. This situation could be sporadic but on the other hand the fact that TP activity is normally very high in platelets^8^ we can speculate that it could be a simple mechanism of compensation and a small laboratory clue towards diagnosis.

One of the remarkable features of our cohort was the identification of the new *TYMP* mutations; p.P131L (c.392 C > T) in nine patients from five separates families and c.978_979ins C which caused a frame shift, in one patient. Screening the proband's parents for p.P131L showed heterozygous carriers for the mutation without clinical features. Family screening identified four individuals with the same homozygous p.P131L mutation, which we refer to as the “Mediterranean” mutation. With the exception of mutation variants (p.V208M)^29^ that retain >14% of healthy control activities of thymidine phosphorylase enzyme activity and was accepted as late‐onset form of MNGIE, generally it was accepted that there was no correlation between genotype and the MNGIE disease phenotype.^1^, ^3^Next to this, based on our experience with nine patients with the “Mediterranean”/homozygous p.P131L (c.392 C > T) mutation we report that the clinical presentation in these patients started fairly early, predominantly with gastrointestinal symptoms and with the disease being rapidly progressive, as shown by no survival beyond 20 years of age. On the other hand, despite the p.P131L variant causing a more severe disease phenotype, an intriguing observation is that the plasma concentrations of dThd and dUrd were in the typical disease range for patient P7 and only mildly elevated in in P6. This particular patient had low concentrations of metabolites but severe clinical findings which emphasizes the importance of not relying on metabolite testing for this particular mutation. We speculate that the genetic background of this patient population may influence the observed disease phenotype. Indeed, there is mounting evidence suggesting that genetic background is as important as the disease‐causing mutation itself due to gene interactions, epigenetics and stochasticity.^30^ Further studies, long term follow‐up and observation on diagnosed patients will be required to elucidate the clinical course and correlation between genotype and phenotype.

Our study had several limitations: because the disease is ultra‐rare the number of number of patients is small and the study is retrospective observational, rather than prospective. However, we believe that, since MNGIE is relatively new disease, and the number of knowledgeable researchers and clinicians is low, the data shared in our paper regarding the t clinical findings and possible prognostic factors could assist clinicians involved in the management of this complex disease.

## FUNDING INFORMATION

No external funding was received for this study.

## CONFLICT OF INTEREST

Sema Kalkan Uçar, Havva Yazıcı, Ebru Canda, Esra Er, Fatma Derya Bulut, Cenk Eraslan, Hüseyin Onay, Bridget Bax and Mahmut Çoker declare that they have no conflict of interest.

## ETHICS STATEMENT

The study was approved by Ege University Medical Faculty ethical committee (E‐99166796‐0500604‐577 754/22‐2.1T/37) and informed concert was obtained from each participant. All procedures followed were in accordance with the ethical standards of the responsible committee on human experimentation (institutional and national) and with the Helsinki Declaration of 1975, as revised in 2000.

## Data Availability

Our manuscript has no associated data.
